# Modifying effect of mouse double minute-2 promoter variants on risk of recurrence for patients with squamous cell carcinoma of oropharynx

**DOI:** 10.1038/srep39765

**Published:** 2017-01-03

**Authors:** Yang Zhang, Erich M. Sturgis, Yuncheng Li, Qingyi Wei, Zhigang Huang, Guojun Li

**Affiliations:** 1 Department of Head and Neck Surgery, The University of Texas MD Anderson Cancer Center, Houston, TX 77030, USA; 2 Department of Otolaryngology Head and Neck Surgery, Beijing Tongren Hospital, Capital Medical University, Key Laboratory of Otolaryngology Head and Neck Surgery, Ministry of Education, Beijing Institute of Otolaryngology, Beijing, 100730, China; 3 Department of Epidemiology, The University of Texas MD Anderson Cancer Center, Houston, TX 77030, USA; 4 Department of Otorhinolaryngology, Union Hospital, Tongji Medical College, Huazhong University of Science and Technology, 1277 Jiefang Avenue, Wuhan 430022, China; 5 Duke Cancer Institute, Duke University Medical Center, Durham, NC 27710, USA

## Abstract

Functional mouse double minute-2 (MDM2) promoter variants may alter *MDM2* expression and thus affect radiotherapy response and prognosis of squamous cell carcinoma of oropharynx (SCCOP). Thus we assessed association of 2 functional *MDM2* promoter variants with recurrence risk of SCCOP. The disease-free survival (DFS) of patients with *MDM2*rs2279744 TT or *MDM2*rs937283 AA genotypes was significantly reduced compared with that of patients with corresponding GT/GG or AG/GG genotypes. Multivariable analysis showed patients with TT or AA genotypes had a significantly higher risk of SCCOP recurrence than those with corresponding GT/GG or AG/GG genotypes did. Furthermore, patients with combined risk genotypes of the 2 polymorphisms had significantly worse DFS and a higher recurrence risk than patients with fewer combined risk genotypes did (*P*_*trend*_ < 0.001). Compared with patients with 0 risk genotypes, patients with 1 or 2 risk genotypes had an approximately 3- or 11-fold increased risk of SCCOP recurrence, respectively. Notably, for both individual and combined polymorphisms, the above similar recurrence risks were particularly higher among patients with human papilloma virus (HPV)-positive tumors. Taken together, our findings suggest that *MDM2* promoter variants individually, or more likely jointly, play a role in determining the risk of recurrence of SCCOP, particularly HPV-positive SCCOP.

Head and neck cancer accounts for less than 5% of all cancers and less 3% of cancer deaths in United States^1^, but is the fifth most common cancer worldwide^2^,^3^. Owing to the continued reduction in tobacco use, the incidence of squamous cell carcinoma of the head and neck (SCCHN), which occurs predominantly in the oral cavity, pharynx, and larynx^4^, has decreased in recent decades^5^,^6^,^7^. In contrast, the incidence of squamous cell carcinoma of the oropharynx (SCCOP), a subtype of SCCHN, is increasing. The increase in SCCOP incidence reflects an increase in human papillomavirus (HPV) infection^8^,^9^. Along with alcohol and tobacco use, HPV is another etiologic agent driving the development of SCCOP^10^. However, only a small proportion of individuals exposed to HPV eventually develop SCCOP, indicating that genetic susceptibility may contribute to an individual’s SCCOP risk^8^.

Although SCCOP treatments have improved recently, the survival of patients with SCCOP has not. One of the major reasons for the poor prognosis of SCCOP is its high recurrence rate, as recurrent SCCOP is associated with poor overall survival^11^,^12^,^13^,^14^. While the recurrence rate might differ among patients with SCCOP, these patients received similar therapeutics and share similar clinical and pathological characteristics. Thus, genetic factors may help determine an individual’s susceptibility to SCCOP recurrence. The identification of new biomarkers that accurately predict SCCOP recurrence would facilitate earlier and improved detection and treatment of the disease. Identifying SCCOP patients at a high risk of recurrence would enable clinicians to quickly select the most appropriate individualized treatment and improve the prognosis of these patients.

The p53 tumor suppressor gene, as “the guardian of the genome,” is a principal mediator of growth arrest, apoptosis, and senescence in response to an array of cellular damages^4^,^15^,^16^. Genetic alterations in p53 have been found in most human cancers^16^. The activity of p53 is negatively regulated by the murine double minute 2 (MDM2) oncoprotein^17^,^18^,^19^,^20^. For example, MDM2 can directly bind to p53 to inhibit its transcriptional activity; quickly enhance the ubiquitination and degradation of p53 through ubiquitin-E3 ligase; and promote p53 degradation by blocking p53’s transport from the nucleus to the cytoplasm^4^,^21^.

Of the *MDM2* promoter polymorphisms identified, 2 single nucleotide polymorphisms (SNPs) of *MDM2, MDM2*rs2279744 and *MDM2*rs937283, have been reported in the risk of SCCHN or HPV-associated oral cancer. However, no large study to date has investigated these SNPs’ associations with the risk of SCCOP recurrence after radiochemotherapy. We hypothesized that functional *MDM2* promoter variants alter the gene’s expression and thus affect the radiotherapy response and prognosis of SCCOP. To test our hypothesis, we used the log-rank test and multivariable Cox models to assess the association of 2 functional *MDM2* promoter variants with recurrence risk in 1008 patients with incident SCCOP.

## Materials and Methods

### Study patients

We identified 1008 patients with SCCOP who were treated with definitive radiotherapy at The University of Texas MD Anderson Cancer Center between May 1995 and April 2010. Patient recruitment, including inclusion and exclusion criteria, has been described previously^22^. Overall, all patients with newly confirmed SCCOP were recruited regardless of age, sex, ethnicity, and clinical disease stages. All patients provided written informed consent to be enrolled in the study, which was approved by The University of Texas MD Anderson Cancer Center’s Institutional Review Board. All informed consent was obtained from all subjects. The committee approved all experiments performed in this study. In addition, all methods were performed in accordance with the relevant guidelines and regulations. All patients were treated with definitive radiation and were followed at our institution. The study outcome was well-defined if patients were considered to be disease-free or to have disease recurrence on the date of last follow-up, disease recurrence, or death. Detailed information about event definition, follow-up, disease stage, treatment, and medical comorbidities, etc., has been described previously^22^. Patients were defined as “ever smokers” or “never smokers” and as “ever drinkers” and “never drinkers” as described previously^22^.

### Genotyping

Genomic DNA extraction with the Qiagen DNA Blood Mini Kit (Qiagen, Valencia, CA) and genotyping for 2 SNPs (*MDM2*rs2279744 and *MDM2*rs937283) were performed as we described previously^23^. For quality control, approximately 10% of samples were randomly selected and re-tested; the results of the repeated tests had a 100% concordance with the results of the original tests.

### Tumor human papillomavirus 16 assay

Tumor human papillomavirus 16 (HPV16) detection was performed using specific polymerase chain reaction and *in situ* hybridization methods as described previously^24^. All patients had paraffin-embedded tumor biopsy or resected tumor specimens available for HPV16 DNA detection. For quality control, we repeated the test in 5% of the samples; the results of repeated tests had a 100% concordance with those of the original tests.

### Statistical analysis

All statistical analyses were performed using SAS software (version 9.2.3; SAS Institute). *P* values less than 0.05 were considered statistically significant in a 2-sided test consideration. The associations between individual epidemiologic risk factors and clinical characteristics and outcome events were estimated using either the Student t-test for continuous variables or the χ^2^ test for categorical variables. Univariate and multivariable analyses with Kaplan-Meier survival estimates, the log-rank test, and Cox proportional hazards regression were performed as described previously^22^. The estimates of the associations between the SNPs and recurrence risk are presented as hazard ratios (HRs) and their 95% confidence intervals (CIs). The Cox regression multivariable analysis was adjusted for important prognostic confounders.

## Results

Of the 1008 SCCOP patients enrolled in the study, 181 had disease recurrence during the follow-up period. The median follow-up time for all patients was 44.7 months (range, 1.7–171.0 months). The median follow-up times for patients with and patients without SCCOP recurrence were 11.6 months and 50.9 months, respectively. Among the 181 patients with SCCOP recurrence, approximately 39% had only distant recurrence, 27% had only local recurrence, 11% had regional recurrence, and 23% had recurrence of more than 1 type. Moreover, of the 432 SCCOP patients whose tumor HPV status was determined, 324 had HPV-positive tumors.

The patients’ characteristics and 5-year recurrence rates are presented in Table 1. Overall, the patients were predominantly non-Hispanic white men. Most patients had late-stage disease and moderate to severe comorbidity. The univariate analyses revealed that age, ethnicity, smoking, alcohol use, comorbidity, and treatment—but not sex or index cancer stage—were significant predictors of DFS (all *P* < 0.05).

**Figure 1:**
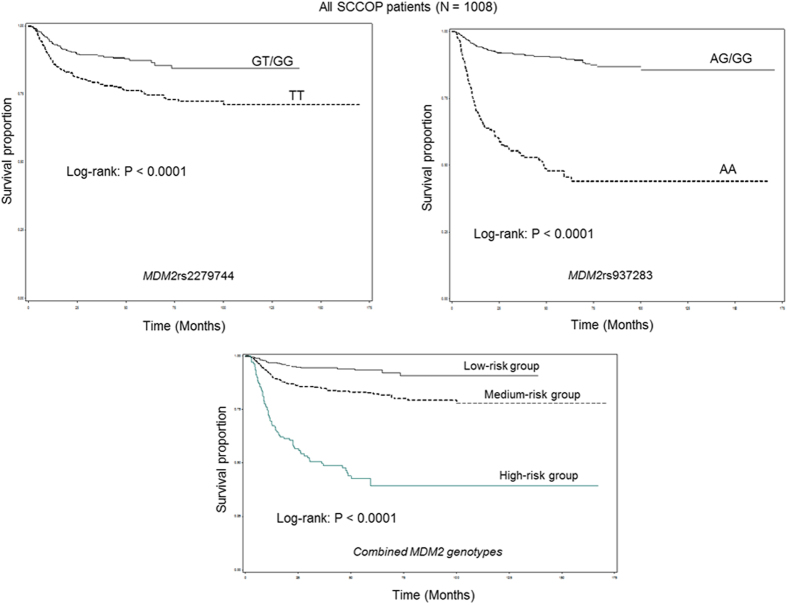
Kaplan-Meier estimates of the disease-free survival of all patients with SCCOP by individual and combined *MDM2* genotypes.

The genotype distributions of the 2 *MDM2* SNPs (*MDM2*rs2279744 and *MDM2*rs937283), the recurrence rates of patients with the SNPs, and the association results are presented in Table 2. Kaplan-Meier analyses revealed that patients with the *MDM2*rs2279744 GT/GG and *MDM2*rs937283 AG/GG genotypes had significantly better DFS than patients with the corresponding TT and AA genotypes did (all log-rank *P* < 0.0001) (Fig. 1). We also categorized the patients into 3 different risk groups based on the number of the combined risk genotypes of the 2 polymorphisms. The low-risk group included patients with no risk genotypes; the medium-risk group included patients with 1 risk genotype of either of the SNPs; and the high-risk group included patients with 2 risk genotypes. These 3 groups had significantly different DFS (log-rank *P* < 0.0001). Furthermore, after adjustment for several major confounders, including age, sex, ethnicity, smoking status, alcohol status, comorbidity, disease stage, and treatment, the multivariable Cox proportional hazards regression analysis showed that the patients with the *MDM2*rs2279744 TT and *MDM2*rs937283 AA genotypes had approximately 2- and 6-fold significantly increased risks of disease recurrence, respectively (aHR, 2.0, 95% CI, 1.5–2.8 and aHR, 6.2, 95% CI, 4.6–8.3, respectively). Table 3 shows the combined effects of the 2 polymorphisms on the risk of recurrence among the 3 different risk groups. A significant trend for increased recurrence risk with increasing number of risk genotypes was observed (*P*_*trend*_ < 0.001); compared with patients in the low-risk group, those in the medium-risk and high-risk groups had approximately 3- and 11-fold increased risks of recurrence, respectively (aHR, 2.7; 95% CI, 1.7–4.2 and aHR, 10.9; 95% CI, 6.8–17.4, respectively). While such stratification of patients is artificial and not based on the statistical results, alternatively, we further categorized the patients into 9 groups based on the combination of risk genotypes of the two polymorphisms as shown in Table 3. As expected, compared with patients with *MDM2*rs2279744 GG and *MDM2*rs937283 GG genotypes, the patients with *MDM2*rs2279744 TT and *MDM2*rs937283 AA genotypes had the highest risk of recurrence among all groups (aHR, 26.8; 95% CI, 10.3–48.7). Additionally, although the patients with *MDM2*rs2279744 GT and *MDM2*rs937283 AG genotypes in the low-risk group, the risk for these patients was higher than those with *MDM2*rs2279744 TT and *MDM2*rs937283 GG genotypes in the medium-risk group. Therefore, such more accurate stratification of patients should be performed instead of the arbitrary method when the larger sample sizes and outcome events of study patients become available.

**Figure 2:**
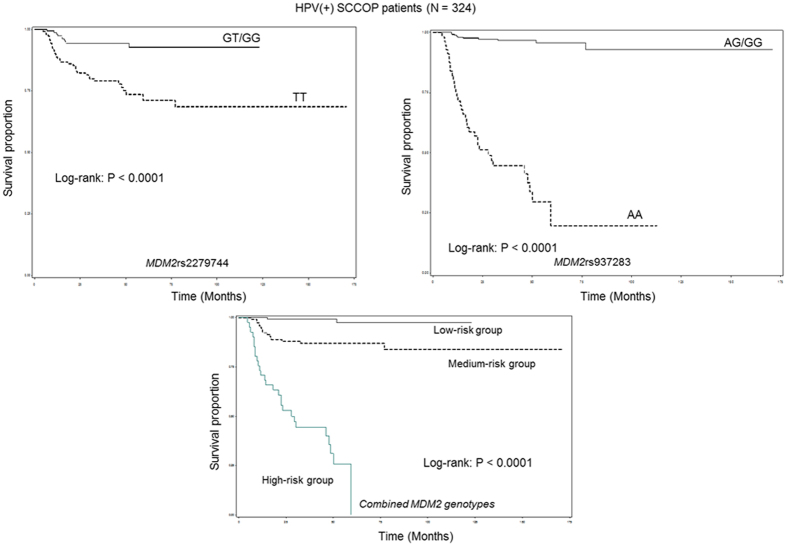
Kaplan-Meier estimates of the disease-free survival of patients with human papillomavirus 16–positive SCCOP by individual and combined *MDM2* genotypes.

Given the roles that HPV and MDM2 have in regulating the p53 pathway, which is important for SCCOP prognosis, we further assessed the associations between the combined SNPs and HPV status and recurrence risk among 324 SCCOP patients with HPV16-positive tumors. As shown in Fig. 2, patients with the *MDM2*rs2279744 GT/GG and *MDM2*rs937283 AG/GG genotypes had significantly better DFS than patients with the corresponding TT and AA genotypes did (all log-rank *P *< 0.0001). A significant difference in DFS was also observed among the 3 different risk groups (log-rank *P* < 0.0001). Multivariable analysis with adjustment for several major confounders showed that the patients with the *MDM2*rs2279744 TT and *MDM2*rs937283 AA genotypes had approximately 4.5- and 24-fold significantly increased risks for recurrence compared with patients with the corresponding GT/GG and AG/GG genotypes (aHR, 4.4, 95% CI, 2.2–8.9 and aHR, 23.7, 95% CI, 11.5–48.8, respectively) (Table 4). Furthermore, as shown in Table 5, the risk of recurrence was even much higher among HPV-positive tumor patients in the medium-risk and high-risk groups (aHR, 10.6, 95% CI, 2.4–46.2 and aHR, 72.7, 95% CI, 17.2–308.6, respectively) when the HPV-positive tumor patients in the low-risk group were used as the reference group. We did not perform a similar analysis for SCCOP patients with HPV16-negative tumors because of the relatively small sample size and the few event outcomes in this subgroup.

## Discussion

In previous studies, we found that the *MDM2*rs2279744 and *MDM2*rs937283 polymorphisms had an interactive effect on HPV-associated SCCOP; moreover, *MDM2*rs2279744 also modified the risk of lung cancer. These finding suggest that these 2 polymorphisms play roles in the etiologies of lung cancers and/or HPV-associated oral cancers^23^,^25^. The present study revealed that both *MDM2*rs2279744 and *MDM2*rs937283 individually, or more likely jointly, significantly increase the risk of SCCOP recurrence after definitive radiotherapy, particularly in patients with HPV-positive tumors. To the best of our knowledge, this is the first study to evaluate associations between these 2 putatively functional MDM2 promoter polymorphisms and the risk of SCCOP recurrence.

The MDM2 protein plays a crucial role in the negative regulation of p53. By inactivating p53, MDM2 overexpression can inhibit p53-mediated tumor-suppressing activities^26^. In addition to interacting with p53, MDM2 also interacts with other key regulatory proteins in molecular pathways that are involved in tumor development and progression and in cancer prognosis, such as cell cycle control, DNA repair, and apoptosis pathways. Therefore, the MDM2 expression level is closely linked with oncogenesis^27^. The 2 *MDM2* SNPs we investigated in the present study have been found to modify cancer development^23^,^25^,^28^,^29^, and our findings may provide some new evidence as to whether these 2 promoter variants of *MDM2* affect recurrence risk among SCCOP patients. The *MDM2-*rs2279744 and *MDM2*rs937283 variants are located in the MDM2 promoter, which initiates differential transcriptions of *MDM2*. In this study, the variant T allele was associated with increased risk of SCCOP recurrence, while others reported that individuals with the *MDM2* G allele have a decreased risk of oral squamous cell carcinoma and leukemia^23^,^30^.

The molecular mechanisms underlying the effects that *MDM2-*rs2279744 and *MDM2*rs937283 genetic variations have on cancer development and progression remain elusive. The rs2279744 variant in the MDM2 promoter causes a T-to-G substitution at the 309 nucleotide site, which increases MDM2’s binding affinity for the transcriptional activator SP1, thereby increasing MDM2 expression and thus enhancing p53 degradation^31^. One recent meta-analysis^32^ investigating the association between positive MDM2 expression and clinicopathological characteristics in patients with esophageal squamous cell carcinoma found that high MDM2 expression was associated with early primary tumor stage and increased risk of regional, but not distant, lymph node metastasis, whereas another study^33^ showed that loss of MTBP (MDM2 binding protein) expression may be linked to worse survival in some patients with SCCHN^32^,^33^. In addition, the interaction between *MDM2* and *p53*, as well as several environmental factors, such as smoking, alcohol use, and HPV infection, may increase the risk of some squamous cell carcinomas^4^,^23^,^34^,^35^,^36^. For example, Chen *et al*. reported that both *MDM2*rs2279744 and *MDM2*rs937283 may synergize with HPV16 L1 seropositivity to significantly increase the risk of oral squamous cell carcinoma, particularly SCCOP^23^.

Many factors, including genetic, epidemiologic, demographic, and clinical variables, may affect SCCOP patients’ risk of recurrence. For example, the risk of SCCOP recurrence could be confounded by tumor HPV status, since HPV-positive SCCOP patients unlikely have somatic genetic changes (e.g., intact p53) compared with HPV-negative SCCOP patients, the majority of whom have disease driven by smoking. Thus, compared with HPV-positive SCCOP patients, HPV-negative SCCOP patients generally have p53 mutations. Owing to such different somatic mutation profiles, these 2 groups may have different responses to radiotherapy. Therefore, HPV status may have an important confounding effect on the prognosis of SCCOP, and stratifying SCCOP patients by tumor HPV status may be necessary to determine prognosis. In the present study, to minimize the confounding effect of tumor HPV status on SCCOP prognosis, we investigated the effects of *MDM2*rs2279744 and *MDM2*rs937283 variants on SCCOP recurrence among HPV-positive patients only. As expected, both the individual- and combined-risk genotypes of these 2 variants had a pronounced modifying effect on SCCOP recurrence risk. The biological mechanisms behind such associations among SCOOP patients remain unclear. We speculate that the *MDM2*rs2279744 and *MDM2*rs937283 risk genotypes may cause p53 degradation, thereby reducing p53-induced apoptotic responses among patients with HPV16-positive tumors, which in turn lead to poor responses to definitive radiotherapy and subsequently an increased risk of disease recurrence. However, these hypotheses need to be verified in future studies.

The present study had some potential limitations. First, the study lacked comprehensive information on the exact dosage and duration of radiotherapy for each patient. Second, because the overall sample size and the number of outcome events of patients with HPV16-positive tumors were relatively small, some significant findings in that group could in fact be due to chance. Thus, our results might not be generalizable and should be confirmed by other larger studies. Third, because most patients in the study were non-Hispanic white patients, our results may not be generalizable to other ethnic groups. Finally, the mechanism underlying the observed associations in the present study is not fully understood, and a more comprehensive study to rule out the competing effects of chance, population stratification, and different genetic backgrounds is warranted.

In conclusion, the genetic promoter variants *MDM2*rs2279744 and *MDM2*rs937283 may play important roles in *MDM2* expression and p53-depenent apoptotic pathways, affect apoptotic capacity and radiotherapy response, and contribute to genetic susceptibility to SCCOP recurrence, particularly among SCCOP patients with HPV16-posiitve tumors who have received definitive radiotherapy. Additional studies to validate and explore the molecular mechanisms underlying the observed associations are needed to assess these SNPs’ utility as clinical prognostic biomarkers.

## Additional Information

**How to cite this article**: Zhang, Y. *et al*. Modifying effect of mouse double minute-2 promoter variants on risk of recurrence for patients with squamous cell carcinoma of oropharynx. *Sci. Rep.* **7**, 39765; doi: 10.1038/srep39765 (2017).

**Publisher's note:** Springer Nature remains neutral with regard to jurisdictional claims in published maps and institutional affiliations.

## Figures and Tables

**Table 1:** Characteristics of 1008 patients with SCCOP.

Variable	No. of patients (%)	No. of patients with recurrence	5-year recurrence rate (%)	P* value
No. of patients	1008 (100)	181	20	
Age, years				
≤57	621 (61.6)	85	15	<0.0001
>57	387 (38.4)	96	27	
Sex				
Male	872 (86.5)	161	20	0.3110
Female	136 (13.5)	20	19	
Ethnicity				
Non-Hispanic white	913 (90.6)	146	17	<0.0001
Other	95 (9.4)	35	41	
Smoking				
Never	388 (38.5)	51	14	0.0004
Ever	620 (61.5)	130	23	
Alcohol use				
Never	247 (24.5)	26	10	0.0005
Ever	761 (75.5)	155	23	
Comorbidity				
None or mild	913 (90.6)	157	19	0.0370
Moderate to severe	95 (9.4)	24	27	
Index cancer stage				
1 or 2	72 (7.1)	11	19	0.5280
3 or 4	936 (92.9)	170	20	
Treatment				
X/XC/XS/S	947 (93.9)	166	19	0.0030
SXC	61 (6.1)	15	32	

X, radiotherapy; C, chemotherapy; and S, surgery.

^*^Log-rank test for disease-free survival between the two groups.

**Table 2:** Association between *MDM2*rs2279744 and *MDM2*rs937283 genotypes and disease recurrence in 1008 patients with SCCOP.

Genotypes	No. of recurrences/no. of patients	5-year recurrence rate, %	Log-rank *P* value	aHR* (95% CI)
*MDM2rs2279744*			< 0.0001	
GT/GG†	52/451	10		1.0
TT	129/557	24		2.0 (1.5–2.8)
*MDM2rs937283*			<0.0001	
AG/GG†	77/797	10		1.0
AA	104/211	52		6.2 (4.6–8.3)

aHR, adjusted hazard ratio; CI, confidence interval.

^*^Adjusted for age, sex, ethnicity, smoking status, alcohol use status, disease stage, comorbidity, and treatment.

^†^Reference group.

**Table 3:** Association between combined risk genotypes of *MDM2*rs2279744 and *MDM2*rs937283 and recurrence risk in 1008 patients with SCCOP.

Combined *MDM2* genotypes	No. of recurrences/no. of patients	5-year recurrence rate, %	Log-rank *P* value	aHR* (95% CI)
Low-risk group†	23/379	6	<0.0001	1.0
Medium-risk group	83/490	17		2.7 (1.7–4.2)
High-risk group	75/139	57		10.9 (6.8–17.4)
Trend test			<0.001	
**Combined** ***MDM2*****genotypes**	**No. of risk genotypes (no. of recurrences/no. of patients)**	**Genotypes of** ***MDM2*** **polymorphisms**		aHR* **(95% CI)**
		rs2279744	rs937283	
1	Low (9/205)	GG†	GG†	1.0
2	Low (5/71)	GG	AG	1.9 (0.3–3.2)
3	Medium (19/192)	GG	AA	3.4 (1.1–5.8)
4	Low (4/67)	GT	GG	1.4 (0.4–4.2)
5	Low (5/36)	GT	AG	2.6 (0.6–5.3)
6	Medium (28/99)	GT	AA	11.2 (6.5–20.1)
7	Medium (4/67)	TT	GG	1.9 (1.0–3.7)
8	Medium (32/132)	TT	AG	7.1 (3.7–13.6)
9	High (75/139)	TT	AA	26.8 (10.3–48.7)

aHR, adjusted hazard ratio; CI, confidence interval.

^*^Adjusted for age, sex, ethnicity, smoking status, alcohol use status, disease stage, comorbidity, and treatment.

^†^Reference group.

**Table 4:** Association between *MDM2*rs2279744 and *MDM2*rs937283 genotypes and recurrence risk in 324 patients with human papillomavirus–positive SCCOP.

Genotypes	No. of recurrences/no. of patients	5-year recurrence rate, %	Log-rank *P* value	aHR* (95% CI)
*MDM2rs2279744*			<0.0001	
GT/GG†	10/178	7		1.0
TT	35/146	26		4.4 (2.2–8.9)
*MDM2rs937283*				
AG/GG†	10/268	4	<0.0001	1.0
AA	35/56	70		23.7 (11.5–48.8)

aHR, adjusted hazard ratio; CI, confidence interval.

^*^Adjusted for age, sex, ethnicity, smoking status, alcohol use status, disease stage, comorbidity, and treatment.

^†^Reference group.

**Table 5:** Association between combined risk genotypes of *MDM2*rs2279744 and *MDM2*rs937283 and recurrence risk in 324 patients with human papillomavirus–positive SCCOP.

Combined *MDM2* genotypes	No. of recurrences/no. of patients	5-year recurrence rate, %	Log-rank *P* value	aHR* (95% CI)
Low-risk group†	2/163	2	<0.0001	1.0
Medium-risk group	16/120	16		10.6 (2.4–46.2)
High-risk group	27/41	74		72.7 (17.1–308.6)
Trend test			<0.001	

aHR, adjusted hazard ratio; CI, confidence interval.

^*^Adjusted for age, sex, ethnicity, smoking status, alcohol use status, disease stage, comorbidity, and treatment.

^†^Reference group.
